# Influence of temperature on sonographic images of the median nerve for the diagnosis of carpal tunnel syndrome: a case control study

**DOI:** 10.1186/s12880-021-00700-6

**Published:** 2021-11-06

**Authors:** Yi-Wei Chang, Chii-Jen Chen, You-Wei Wang, Valeria Chiu, Shinn-Kuang Lin, Yi-Shiung Horng

**Affiliations:** 1 Department of Physical Medicine and Rehabilitation, Taipei Tzu Chi Hospital, Buddhist Tzu Chi Medical Foundation, No. 289, Jianguo Rd., Xindian Dist., New Taipei City, 231 Taiwan, ROC; 2 Department of Medicine, Tzu Chi University, Hualien, Taiwan, ROC; 3 Department of Medical Imaging and Radiological Technology, Yuanpei University of Medical Technology, Hsinchu, Taiwan, ROC; 4 Department of Computer Science and Information Engineering, National Taiwan University, Taipei, Taiwan, ROC; 5 Stroke Center and Department of Neurology, Taipei Tzu Chi Hospital, Buddhist Tzu Chi Medical Foundation, New Taipei City, Taiwan, ROC; 6 Graduate Institute of Injury Prevention and Control, College of Public Health, Taipei Medical University, Taipei, Taiwan

**Keywords:** Carpal tunnel syndrome, Ultrasonography, Power Doppler, Skin temperature, Blood flow

## Abstract

**Background:**

In addition to nerve conduction studies (NCSs), ultrasonography has been widely used as an alternative tool for diagnosing carpal tunnel syndrome (CTS). Although the results of NCSs are influenced by local skin temperature, few studies have explored the effects of skin temperature on ultrasonography of the median nerve. Since swelling and intraneural blood flow of the median nerve might be influenced by local temperature changes, the aim of this study was to evaluate the cross-sectional area (CSA) and intraneural blood flow of the median nerve under three skin temperatures (30 °C, 32 °C, 34 °C).

**Methods:**

Fifty patients with CTS and 50 healthy volunteers were consecutively recruited from a community hospital. Each participant received physical examinations and NCSs and underwent ultrasonography, including power Doppler, to evaluate intraneural vascularity.

**Results:**

The CSA of the median nerve in the CTS patients was significantly larger than that in the healthy controls at all three temperatures. However, significant differences in the power Doppler signals of the median nerve between the two studied groups were observed only at 30 and 32 °C, not at 34 °C.

**Conclusion:**

The significant difference in the intraneural vascularity of the median nerve between the patients with CTS and the healthy subjects was lost at higher temperatures (34 °C). Therefore, the results of power Doppler ultrasonography in diagnosing CTS should be cautiously interpreted in patients with a high skin temperature or those who reside in warm environments.

## Introduction

For decades, ultrasound examination has gained increasing popularity as a useful tool to evaluate patients with carpal tunnel syndrome (CTS). Several ultrasonography signs of CTS have been proposed, such as an increase in the cross-sectional area (CSA) of the median nerve, an increased palmar bowing of the flexor retinaculum, and an increased flattening ratio (the ratio of the long axis of the median nerve to the short axis) [1]. Among the various diagnostic criteria, the CSA of the median nerve at the wrist level was shown to achieve a sensitivity of 65% to 97%, specificity of 72.7% to 98%, and accuracy of 79% to 97%, which was considered to be as accurate as electrodiagnostic studies [2]. Compared with other imaging studies, such as MRI examination, ultrasonography has been shown to be an adequate screening method for CTS [3].

However, using a single cutoff value for the CSA of the median nerve has been argued to underestimate the prevalence rate of CTS, given the variations in body weight, age, and sex among individuals [4, 5]. Therefore, alternative methods have been proposed for ultrasound examination of CTS patients. For example, comparative methods such as the swelling ratio and ulnar to median ratio use the median nerve at the forearm level or ulnar nerve at the wrist level as an internal control to improve the diagnostic accuracy [4, 6–9]. Using power Doppler ultrasonography to evaluate the intraneural vascularity of the median nerve, on the other hand, has also emerged in recent years as another method to assist in diagnosing CTS [10, 11]. The sensitivity and specificity of intraneural vascularity in diagnosing CTS have also been reported to be similar to those of electrodiagnostic studies [12]. For example, Dejaco, et al. semiquantitatively graded intraneural power Doppler signals as a score of 0 to 3, and the results showed that a score of 2 (two or three single vessels or two confluent vessels within the median nerve) or above had a specificity of 90% for the diagnosis of CTS [10]. One recent study calculated the total area of the vascularity on power Doppler images of each median nerve, and the results showed that the sensitivity and specificity of CSA and hypervascularization in detecting CTS were 90.9, 94.0, 93.4, and 90.0%, respectively [11].

Regarding the diagnosis of CTS by nerve conduction studies (NCSs), skin temperature is considered a crucial factor, as previous studies have shown that nerve conduction velocity is affected by skin temperature. Burnham et al. showed that increasing the hand temperature up to 33.5 °C by using a heating pad for 20 minutes resulted in significantly faster transcarpal tunnel median nerve sensory and motor conduction, thus leading to 15% fewer limbs meeting the NCS diagnostic criteria of CTS [13]. Therefore, it is recommended to monitor and maintain the hand temperature at 32–33 °C while performing NCS examinations [14, 15]. Although several ultrasonographic studies have shown increased intraneural vascularity of the median nerve in patients with CTS [10, 11, 16], none of them have examined the effect of skin temperature change on the CSA and intraneural vascularity of the median nerve. Since median nerve swelling and its intraneural blood flow might be influenced by local temperature changes, the aim of this study was to evaluate the CSA of the median nerve and intraneural vascularity at three skin temperatures (30 °C, 32 °C, 34 °C). We also evaluated blood flow changes in the ulnar artery as validation of the effect of skin temperature changes on local blood flow.

## Materials and methods

### Patients

We recruited patients with CTS from the Physical Medicine and Rehabilitation department of a community hospital. The control participants were recruited among healthy volunteers, hospital staff, and their family or friends. This study was approved by our Institutional Review Board, and informed consent was obtained from each participant. All methods in the study were carried out in accordance with the guidelines outlined in the Declaration of Helsinki.

Patients with CTS presenting with the following symptoms were included: numbness, paresthesia, or tingling pain over the median nerve-innervated area of the involved hand. Furthermore, the patients were required to have positive responses in either Tinel’s test or Phalen’s test during physical examination and had to show evidence of median neuropathy at the wrist level in the NCS. The control participants could not exhibit symptoms or signs of CTS and had no abnormal findings in the electrodiagnostic study of the median and ulnar nerves across the wrist joint [17]. The patients with CTS and the control participants were excluded if the following exclusion criteria were met: pregnancy; age < 18 years; previous history of hand injury or surgery; medical history of diabetes mellitus, uremia, rheumatoid arthritis, hypothyroidism, amyloidosis, or acromegaly. Clinical examinations for each participant included a baseline survey, a physical examination, an NCS, and an ultrasound examination.

### Physical examination

While performing Phalen’s test, the participants were asked to hold both wrists in a full flexion position for 60 seconds. Patients experiencing characteristic CTS symptoms (tingling, paresthesia, and numbness in the median nerve-innervated area) were regarded as having a positive response. During Tinel’s test, the median nerve was tapped along its course through the carpal tunnel and a positive result was defined as the elicitation of characteristic CTS symptoms [18].

The Semmes–Weinstein monofilament sensory test was carried out by applying the force-calibrated monofilament perpendicularly to the volar digital surface, and the pressure was increased until the monofilament began to bend. The participants were asked to close their eyes, and a normal response was recorded when they could identify which finger was tested based on one out of three responses to the 2.83 g monofilament, while a positive response to higher than 2.83 g indicated “diminished” sensation. A weighted score from 1 to 5 was given depending on the calculated force of each filament, and a lower score indicated greater force [19]. We measured grip strength using a handheld dynamometer and asked each participant to perform three recorded trials and recorded the mean score [20].

### Nerve conduction study

We performed an NCS for each participant using Neuropack M1 MEB-9200 J/K electrodiagnostic equipment (Nihon Kohden Corporation, Tokyo, Japan) in a quiet, air-conditioned room with the ambient temperature maintained at 26 °C. Each participant was placed in a supine position, and the temperature of each hand was maintained at ≥ 32 °C. NCS was conducted using the supramaximal stimulation technique. We measured distal motor latency of the median nerve by placing a stimulating electrode at the wrist and a recording electrode 7 cm away from the stimulating electrode on the abductor pollicis brevis muscle [14, 21]. For sensory NCSs of the median nerve, the distance between the stimulating electrode at the wrist and the recording electrodes at the index finger was maintained at 14 cm. A short segment study of palmar median mixed nerve was conducted by placing a stimulating electrode on the web space between the second and third metacarpal bone, 8 cm from the recording electrode at the wrist.

The clinical diagnosis of CTS was confirmed if one of the following criteria was met in the median nerve NCS: (1) distal motor latency > 4.4 ms, (2) distal sensory latency > 3.5 ms, or (3) midpalm median nerve peak latency > 2.2 ms [2, 14].

### Ultrasound examination

Ultrasound examination was performed using the GE logic P6 device (General Electric Medical Systems, Milwaukee, WI, USA) with an 11 L linear array transducer (12 MHz). The participants were asked to lie on the bed in a supine position with the elbow extended, forearm supinated, and wrist and hand in the neutral position in a quiet, air-conditioned room with the ambient temperature maintained at 26 °C. The transducer was placed perpendicular to the surface of the median nerve under examination using a transverse scanning technique. Care was taken to avoid applying additional forces on the transducer during the entire scanning process to prevent further nerve deformation [17]. Ultrasonography was performed by one physiatrist who was board certified in musculoskeletal ultrasonography for more than five years and blinded to the clinical and NCS findings.

Skin temperature was continuously monitored using a digital skin thermometer (TK-62, New Taipei city, Taiwan) with the electrode fixed at the palmar area during the whole procedure. We applied an ice pack first to each subject’s hand and distal part of the forearm until the skin temperature decreased to 30 °C and then performed ultrasonography, including (1) B mode ultrasonography to evaluate the CSA of the median nerve at the pisiform level; (2) power Doppler ultrasonography to evaluate the intraneural vascularity of the median nerve at the distal crease level (capitate-lunar junction); and (3) spectral Doppler ultrasonography to evaluate blood flow of the ulnar artery at the distal crease. After finishing the above ultrasonography at 30 °C, we applied a hot pack to each subject’s hand and distal part of the forearm until the skin temperature increased to 32 °C and performed the same ultrasonography protocol again. The skin temperature usually remained at the target temperature for approximately 2 min, so we had sufficient time to finish the ultrasonography. After that, a hot pack was further applied until the skin temperature reached 34 °C, and B mode scan and power and spectral Doppler examinations were repeated for each hand.

### Acquisition of B mode transverse ultrasonogram

**Fig. 1:**
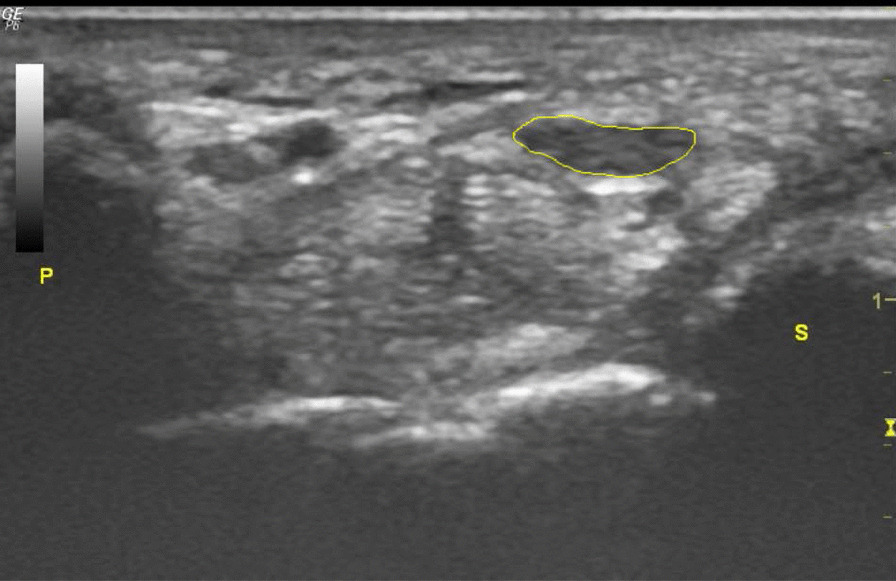
Cross-sectional area of the median nerve (encircled area) at the pisiform level; P: pisiform bone; S: scaphoid bone

Transverse ultrasonograms were obtained at the level of pisiform bone. We used electronic calipers to measure the CSA of the median nerve by tracing the margin of the inner border of the perineural hyperechogenic rim that surrounded the hypoechoic median nerve [1, 11] (Fig. 1).

### Acquisition of power Doppler and spectral Doppler ultrasonograms

After finishing transverse ultrasonography, the transducer was kept longitudinal to the surface of the median nerve above the distal wrist crease. Power Doppler ultrasonography was applied to the grayscale image and optimized using a standardized set of technical settings (frequency: 6.7 MHz, pulse repetition frequency: 600 Hz). The power Doppler color box was restricted to the median nerve at the level of the distal wrist crease, and the power Doppler sonogram was obtained at 30 °C, 32 °C and 34 °C. Vascularity was measured for the median nerve within the borders of the epineurium. Moreover, we recorded the power signal three times consecutively and kept the pressure of the probe at a minimum to prevent the obliteration of small vessels.

**Fig. 2:**
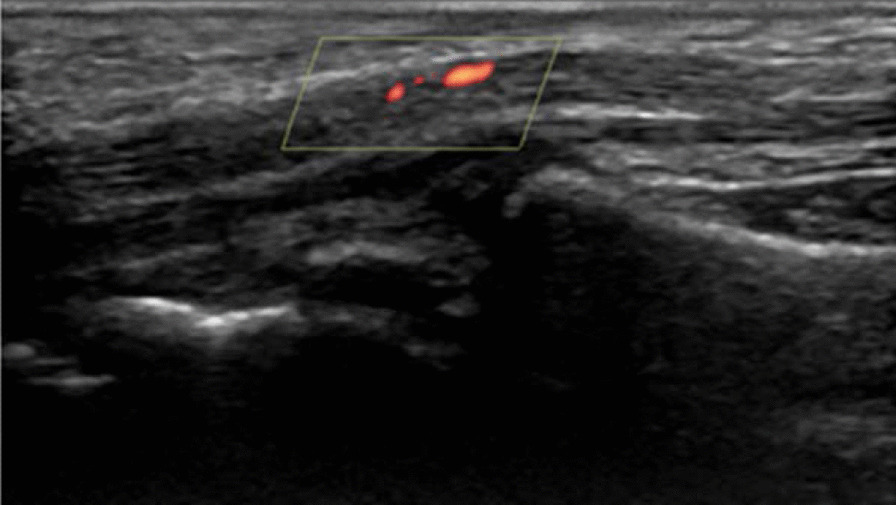
Intraneural blood flow of median nerve depicted by power Doppler at the distal crease level

To quantitatively analyze the region of intraneural blood flow shown in the power Doppler results (Fig. 2), the image was processed using a customized segmentation algorithm designed by coauthors Chen and Wang. We selected the region of interest (ROI) and used a power region extraction framework based on HSV (hue, saturation, value) color space to determine the specific color area in the intraneural blood flow region [22]. Then, we calculated this area of intraneural blood flow in mm^2^ units as one pixel equal to 0.0048 mm^2^. The details of this computer-aided algorithm are described in the “Appendix”.

Blood flow changes in the ulnar artery at three skin temperatures (30 °C, 32 °C and 34 °C) were evaluated by pulsed spectral Doppler while the transducer was kept longitudinal to the ulnar artery. The time-averaged mean velocity (TAMEAN) and resistive index (RI) were recorded. RI is defined as the difference between the peak systolic velocity and end diastolic velocity divided by the peak systolic velocity [23].

### Statistical analysis

We used Student’s t-test and the chi-squared test to compare the demographic data between the CTS patients and the healthy volunteers. Considering that some tests/examinations were performed for both hands of bilateral CTS patients and healthy volunteers, a generalized estimating equation (GEE) adjusting for age and sex was used to compare the above results between the two studied groups. Repeated-measures ANOVA (analysis of variance) was used to compare results among three different temperatures (30 °C, 32 °C, 34 °C), including B mode and power Doppler findings of the median nerve and spectral Doppler results of the ulnar artery. All of the statistical analyses were performed using SAS Version 9.2 (SAS Institute Inc., Cary, NC, USA).

## Results

Fifty patients with CTS who had either a positive Tinel or Phalen sign with positive NCS findings were consecutively recruited. Among these patients, 34 had bilateral CTS. Fifty age- and sex-matched healthy volunteers were also recruited. As shown in Table 1, no significant differences in demographic data were found between the patients and the healthy volunteers except employment status and education level. We excluded 16 uninvolved hands in the CTS group and 3 asymptomatic hands with abnormal NCS findings in the healthy group; therefore, 84 CTS hands and 97 healthy hands were included in the final analyses.

**Table 1:** Frequency distribution of demographic data for patients with patients with carpal tunnel syndrome (CTS) and healthy volunteers

Characteristics	CTS patients (n = 50) N (%)	Healthy volunteers (n = 50) N (%)	*p* value^a^
Age, mean ± SD, yrs	50.9 ± 10.0	47.8 ± 9.6	0.15
*Personal characteristics*			
Female	44 (88.0)	44 (88.0)	1.00
Married	38 (76.0)	34 (70.8)	0.56
Employed	25 (52.1)	37 (74.0)	0.03
Smoking habit	2 (4.0)	1 (2.0)	1.00
Right-hand dominant	46 (97.9)	48 (98.0)	1.00
Unilateral hand involved	16 (32.0)	–	
Bilateral hands involved	34 (68.0)	–	
*Educational level*			
College/University	30 (60.0)	43 (86.0)	0.0033
Senior high	14 (28.0)	2 (4.0)	
Junior high or below	6 (12.0)	5 (10.0)	

^a^*p* value, comparison between CTS patients and healthy volunteers by Student *t* or chi-square test

Significant differences in the monofilament sensory test and NCS were found between the CTS and healthy groups (Table 2). There were also significant differences between the two studied groups in the CSA of the median nerve measured at 30, 32 and 34 °C. The area of intraneural vascularity of the median nerve on power Doppler images was also significantly larger in the CTS patients than the healthy volunteers at 30 and 32 °C but not at 34 °C (1.13 vs 0.65 mm^2^, *p* = 0.07) (Table 3). In comparison, among the within-group results measured at 30, 32 and 34 °C in both studied groups, no significant differences were found in the sonographic findings of the median nerve, including the CSA and the power Doppler signal (Table 3). Regarding the spectral Doppler findings of the ulnar artery, at 30, 32 and 34 °C, significant differences were found in RI and TAMEAN in the CTS patients and in the healthy volunteers. No significant difference was found in RI and TAMEAN between the studied groups at any of the three temperatures (Table 4).

**Table 2:** Comparison of results of physical examinations and nerve conduction studies between hands with carpal tunnel syndrome (CTS) and healthy hands

Variables	CTS hands (n = 84)	Healthy hands (n = 97)	*p* value^a^
	Mean ± SD	Mean ± SD	
*Physical examination*			
Monofilament sensory test	31.9 ± 2.9	33.6 ± 1.8	0.0003
Grip strength (kg)	48.2 ± 17.7	54.2 ± 18.8	0.07
*Nerve conduction study*			
Distal sensory latency of median nerve (ms)	4.3 ± 3.4	2.9 ± 0.4	0.001
Distal motor latency of median nerve (ms)	5.1 ± 1.3	3.5 ± 0.4	< 0.0001
Midpalm median nerve peak latency (ms)	2.8 ± 0.8	1.8 ± 0.4	< 0.0001

^a^Comparison of differences between groups after adjusting for age and gender (generalized estimating equation)

**Table 3:** Comparison of sonographical findings between hands with carpal tunnel syndrome (CTS) and healthy hands

Variables	Patients with CTS (hands = 84)		Healthy volunteer (hands = 97)		*p* value^a^
	Mean	SD	Mean	SD	
*CSA of median nerve (mm*^*2*^*)*					
30 °C	11.22	4.15	8.92	2.30	< 0.0001
32 °C	11.16	4.38	8.73	2.17	< 0.0001
34 °C	11.48	4.52	8.94	2.31	< 0.0001
*p* value^b^	0.73		0.38		
*Intraneural vascularity area of median nerve by power Doppler (mm*^*2*^*)*					
30 °C	1.23	2.21	0.35	0.86	0.003
32 °C	1.49	2.62	0.55	1.21	0.01
34 °C	1.13	1.93	0.65	1.42	0.07
*p* value^b^	0.33		0.24		

^a^Comparison of differences between groups after adjusting for age and gender (generalized estimating equation)

^b^Comparison of differences within group among 30 °C, 32 °C, 34 °C (repeated measures analysis of variance)

CSA, Cross-sectional area

**Table 4:** Comparison of Doppler findings of ulnar artery between hands with carpal tunnel syndrome (CTS) and healthy hands

Variables	Patients with CTS (hands = 84)		Healthy volunteer (hands = 97)		*p* value^a^
	Mean	SD	Mean	SD	
*Resistive index*					
30 °C	0.95	0.12	0.94	0.17	0.94
32 °C	0.9	0.13	0.91	0.15	0.36
34 °C	0.88	0.17	0.89	0.14	0.40
*p* value^b^	0.0039		0.0350		
*TAMEAN (cm/s)*					
30 °C	5.26	3.55	4.61	3.49	0.37
32 °C	4.27	3.90	3.60	3.10	0.32
34 °C	6.86	4.52	6.86	4.60	0.87
*p* value^b^	< 0.0001		< 0.0001		

TAMEAN, Time-averaged mean velocity

^a^Comparison of differences between groups after adjusting for age and gender (generalized estimating equation)

^b^Comparison of differences within group among 30 °C, 32 °C, 34 °C (repeated measures analysis of variance)

## Discussion

Our data showed that even when the skin temperature changed from 30 to 32 to 34 °C, the CSAs of the median nerve did not show significant changes in either studied group; moreover, they remained significantly larger in patients with CTS than in healthy controls. This finding demonstrates that CSA of the median nerve is a robust criterion in diagnosing CTS, even under different skin temperatures. On the other hand, we found significant differences in the power Doppler signals of the median nerve between the two studied groups measured at 30 and 32 °C but not at 34 °C. Therefore, our results not only are consistent with previous studies [10, 11] but also show that patients with CTS have significantly higher vascularity of the median nerve than healthy controls at skin temperatures of 30 °C and 32 °C. To the best of our knowledge, this is the first study to investigate the effects of different skin temperatures on the results of ultrasonography examination in patients with CTS.

Although no consensus on the pathophysiology of the increased vascularity in the median nerve has been made, it has been proposed that patients with CTS have an increased intraneural vascularity of the median nerve at the carpal tunnel proximal to the entrapment site, probably due to the compensatory effect of distal ischemia [16]. Compressive neuropathy at the wrist might progress through three phases: the first stage is venous congestion of the median nerve, followed by the second stage of median nerve edema and the final stage of impairment in the arterial and venous supply [24]. A previous study showed that unmyelinated sympathetic nerve fibers are located in the periphery of the median nerve trunk and are vulnerable to compression damage or increased carpal tunnel pressure [23]. Vasomotor dysfunction might be another mechanism of increased intraneural vascularity in CTS patients. Wilder-Smith et al. demonstrated that hands with CTS have significantly reduced vasoconstriction in the third and fourth digits, confirming that vasomotor dysfunction mostly follows along the conventional median nerve territories of the hand [25]. Some researchers have also considered that tenosynovitis of the flexor tendons inside the carpal tunnel might lead to abnormal release of vascular endothelial growth factor and prostaglandin E2, which mediate vasodilatation and angiogenesis [26]. Moreover, CTS may not only be a peripheral nerve disorder but also might be accompanied by maladaptive cortical neuroplasticity. For example, Maeda, Y., et al. demonstrated significant gray matter reductions in the contralesional primary somatosensory cortex (hand), pulvinar and frontal pole on magnetic resonance imaging (MRI) scans in CTS patients compared to healthy controls [27]. Event-related functional MRI also revealed that CTS patients had a reduced second/third interdigit cortical separation distance in the contralateral primary somatosensory cortex [28]. Therefore, chronic repetitive painful peripheral stimuli might induce plastic changes in the central nervous system that could lead to central sensitization. This might be the reason why some patients with CTS show symptoms beyond the median nerve innervated dermatome, such as the forearm [29].

Low temperature is considered one of the contributing factors for CTS. Workers in a frozen food factory with hand exposure to a cold environment have a higher risk of developing CTS [30]. Another study showed that working in a cold environment had a higher odds ratio (OR) for developing CTS (OR 3.52, 95% CI 1.08–11.47) than working with repeated movements of the wrist (OR 2.15, 95% CI 1.14–4.07) [31]. Araujo et al. also reported that after immersion in ice water, CTS patients had a more significant change in distal sensory latency of the median nerve than healthy subjects, which implied that CTS patients are more sensitive to cold exposure and tend to have Raynaud’s phenomenon [32].

In this study, we demonstrated that there was no significant difference in the RI or TAMEAN of the ulnar artery measured by spectral Doppler ultrasound between patients with CTS and control participants, which was consistent with previous findings in CTS patients with diabetes [33]. Furthermore, our study showed that as the skin temperature rose from 30 to 34 °C, there was a gradual decrease in the RI and an increase in the TAMEAN of the ulnar artery within each group. RI is an indicator that reflects vascular wall extensibility and the related resistance [34]. Thus, our findings imply that the higher the hand temperature is, the lower the resistance of blood flow; as a result, the blood flow velocity of the ulnar artery gradually increased in both studied groups.

However, to our surprise, we also found that when skin temperature was increased from 32 to 34 °C, the power Doppler signal inside the median nerve did not increase in the CTS patients as in the healthy subjects but rather decreased, although this decrement was not statistically significant. A possible explanation of this phenomenon is that we applied hot packs over the hand and distal part of the forearm. Therefore, when the skin temperature was heated to 34 degrees, increased blood circulation improved venous congestion inside the median nerve of the CTS patients, thus diminishing the power Doppler signal in the patient group. This might be the reason why significant differences were found between patients with CTS and healthy controls at 30 and 32 °C but not at higher temperatures (34 °C). These results coincide with previous NCS findings showing that when skin temperature is elevated, sensory nerve conduction velocity is increased and distal motor latency of the median nerve is decreased, which would lead to false negatives in diagnosing CTS [15]. Therefore, when using power Doppler ultrasonography to aid in CTS diagnosis, skin temperature should be monitored and kept below 34 °C to prevent undermining the diagnostic ability of power Doppler examination. Additionally, the power Doppler results should be cautiously interpreted in CTS patients with high skin temperatures.

Our study is not without limitations. First, this study was performed in a Physical Medicine and Rehabilitation department, so the patients usually suffered from mild to moderate symptoms. Therefore, we should remain cautious in our attempts to generalize our findings to patients with more severe symptoms. Second, we only recruited patients with idiopathic CTS, which might not be reflective of the reality of daily clinical practice, where patients may have median neuropathy at the wrist level that may be secondary to various types of underlying conditions. Thus, future study of CTS patients with comorbidities, such as diabetes and chronic renal failure, is recommended. Third, using power Doppler ultrasonography to detect intraneural vascularity within the median nerve is sensitive to motion artifacts. Thus, we held the probe still and recorded the power signal three times consecutively.

## Conclusions

Although the median nerve CSA remained unaltered under three different temperatures (30 °C, 32 °C, 34 °C), there was a significant difference between patients with CTS and healthy subjects. However, the significant difference in the intraneural vascularity of the median nerve between patients with CTS and healthy subjects was lost at higher temperatures (34 °C). Therefore, the results of power Doppler ultrasonography in patients with CTS should be cautiously interpreted in patients with high skin temperature or those who reside in warm environments.

## Figures and Tables

**Fig. 3:**
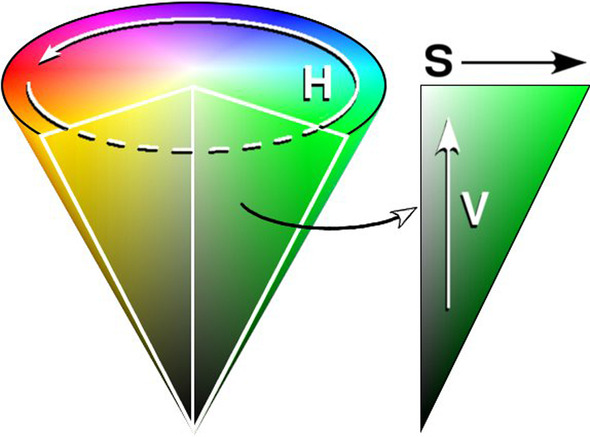
HSV color space model

**Fig. 4:**
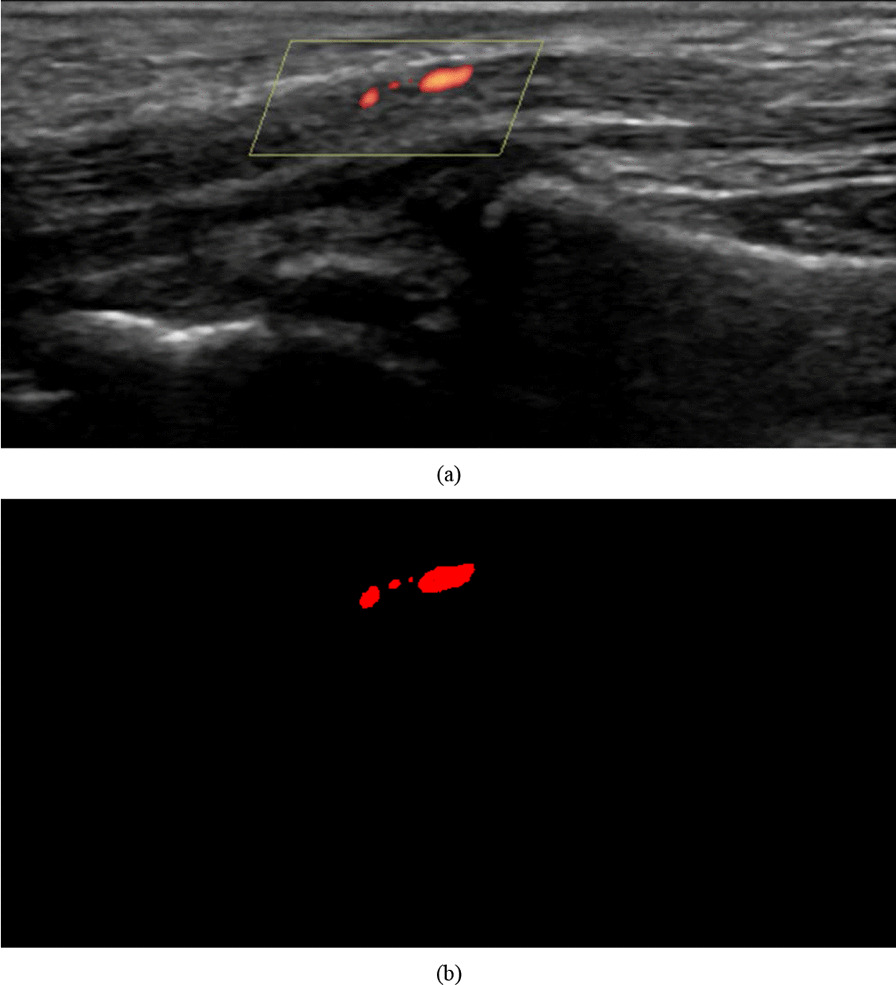
The result after HSV color separation. **a** Shows power Doppler used on US images; **b** is the segmentation results

## Data Availability

All the data needed to achieve the conclusion are contained within the paper. The raw data that support the findings of this study are available from the corresponding author, [YSH], upon reasonable request.

## Appendix

### Power Doppler image segmentation based on HSV color space

We separate the color to perform area calculations in power Doppler images using the HSV (Hue, Saturation, Value) color space model developed and validated by Joblove and Greenberg (Joblove and Greenberg 1978). When the color space transfers to HSV, it is easy to discriminate what color in the power Doppler image. Figure 3 shows the HSV color space model. Hue (H) is a primary attribute that can also present the color from 0° to 359°. For example: red, yellow, green and blue. Saturation (S) was defined as pure color in the range of 0% (gray) to 100% (colorful). Value (V) is the lightness or darkness of the color.

In the power Doppler image, we calculated the area of the blood flow through the median nerve in different red colors with a standard color bar. We focus on the red and the color closest to red to use HSV color space to segment the color in the target region. The threshold setting of the HSV color space model is shown in Table

**Table 5:** Color classification

Color	Range
*When S* *>* *0.3 and V* *>* *0.3*	
Red	H < 30 or H ≥ 330
Yellow	30 ≤ H < 90
Green	90 ≤ H < 150
Cyan	150 ≤ H < 210
Blue	210 ≤ H < 270
Magenta	270 ≤ H < 330
*When S* *≤* *0.3*	
Black	V < 0.5
White	V ≥ 0.5

^5^. In the procedure, we choose the thresholds of red and magenta in Table 5, and the segmentation results are shown in Fig. 4. The results show that the HSV color space efficiently separates color in power Doppler images with IOUs (intersection over union) higher than 0.9.

## Abbreviations

ANOVA: Analysis of variance
AUC: Area under the curve
CSA: Cross-sectional area
CTS: Carpal tunnel syndrome
GEE: Generalized estimate equation
MRI: Magnetic resonance imaging
NCS: Nerve conduction study
OR: Odds ratio
RI: Resistive index
ROC: Receiver operator characteristic
ROI: Region of interest
TAMEAN: Time-averaged mean velocity
US: Ultrasonography
